# Identifying animal behaviours from accelerometers: Improving predictive accuracy of machine learning by refining the variables selected, data frequency, and sample duration

**DOI:** 10.1002/ece3.11380

**Published:** 2024-05-16

**Authors:** Carolyn E. Dunford, Nikki J. Marks, Rory P. Wilson, D. Michael Scantlebury

**Affiliations:** ^1^ School of Biological Sciences Queen's University Belfast Belfast UK; ^2^ Panthera New York City New York USA; ^3^ Biosciences Swansea University Swansea UK

**Keywords:** activity budget, biologging, domestic cat, machine learning, predictive accuracy, random forest model

## Abstract

Observing animals in the wild often poses extreme challenges, but animal‐borne accelerometers are increasingly revealing unobservable behaviours. Automated machine learning streamlines behaviour identification from the substantial datasets generated during multi‐animal, long‐term studies; however, the accuracy of such models depends on the qualities of the training data. We examined how data processing influenced the predictive accuracy of random forest (RF) models, leveraging the easily observed domestic cat (*Felis catus*) as a model organism for terrestrial mammalian behaviours. Nine indoor domestic cats were equipped with collar‐mounted tri‐axial accelerometers, and behaviours were recorded alongside video footage. From this calibrated data, eight datasets were derived with (i) additional descriptive variables, (ii) altered frequencies of acceleration data (40 Hz vs. a mean over 1 s) and (iii) standardised durations of different behaviours. These training datasets were used to generate RF models that were validated against calibrated cat behaviours before identifying the behaviours of five free‐ranging tag‐equipped cats. These predictions were compared to those identified manually to validate the accuracy of the RF models for free‐ranging animal behaviours. RF models accurately predicted the behaviours of indoor domestic cats (F‐measure up to 0.96) with discernible improvements observed with post‐data‐collection processing. Additional variables, standardised durations of behaviours and higher recording frequencies improved model accuracy. However, prediction accuracy varied with different behaviours, where high‐frequency models excelled in identifying fast‐paced behaviours (e.g. locomotion), whereas lower‐frequency models (1 Hz) more accurately identified slower, aperiodic behaviours such as grooming and feeding, particularly when examining free‐ranging cat behaviours. While RF modelling offered a robust means of behaviour identification from accelerometer data, field validations were important to validate model accuracy for free‐ranging individuals. Future studies may benefit from employing similar data processing methods that enhance RF behaviour identification accuracy, with extensive advantages for investigations into ecology, welfare and management of wild animals.

## Graphical abstract

Machine learning offers a robust means of behaviour identification from accelerometer data. However, simple data processing, including calculating additional descriptive variables, altering data frequencies and standardising durations of behaviours in the training dataset, enhances predictive accuracy by random forest models. Observations are important to validate behaviour predictions for free‐ranging individuals but can provide robust behaviour identifications and enhance investigations into ecology, welfare and management of wild animals.

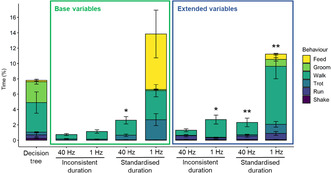

## 1 INTRODUCTION

Animal‐attached tri‐axial accelerometer loggers, which measure both gravitational and inertial acceleration at high frequency, provide a useful means of recording wild animal behaviours (Gooden et al., 2024; Shepard, Wilson, Quintana, et al., 2008; Wilmers et al., 2015) as well as those of animals in captivity and agriculture (Alvarenga et al., 2016; Hathaway et al., 2023). This informs many aspects of species' biology such as their ecology and movements (Bidder et al., 2020; Ullmann et al., 2023), energetics (Dunford et al., 2020; Pagano & Williams, 2019), diel activity patterns (Bryce et al., 2022; Migli et al., 2021), conservation and management (McGowan et al., 2022; Wijers et al., 2018) and welfare (Barbour et al., 2019; Soltis et al., 2012).

Classification of behaviours from acceleration data can be achieved manually, through observing animals and attributing acceleration signals to different behaviours undertaken (Wilson et al., 2006). Decision trees then utilise a series of questions to categorise the data with respect to the observed signal criteria (McClune et al., 2014; Riaboff et al., 2019; Valletta et al., 2017). Although decision trees can be accurate and effective, they are time‐consuming to construct and use, especially when animals are monitored for long periods of time and undertake many different behaviours (Hammond et al., 2016). Increasingly, machine learning is being used to automate behaviour recognition, either through unsupervised or supervised methods. Unsupervised machine learning groups acceleration signals into likely behaviour categories by identifying similarities in patterns. More commonly, supervised machine‐learning methods, such as random forest (RF) models, are trained using previously classified accelerometer data and are then used to predict animal behaviours using distinct accelerometer attributes (Breiman, 2001). These methods can rapidly and accurately identify vast datasets from animal behaviours in the wild, where observation is not always possible.

Accelerometer data calibrated via observations forms a behaviour ‘training’ dataset (Shuert et al., 2018; Wang, 2019). RF models generate multiple (e.g. 300+) decision trees, and the most frequent predicted classification from the many individual trees generated is selected as the predicted behaviour for each time period (Li, 2013). Training datasets are generated from a proportion of the training data (60%–80%), which can be tested for predictive accuracy using the remaining test data (Lush et al., 2016; Venter et al., 2019). Validation using data that was not initially used to train the model provides an independent measure of predictive accuracy.

Overall, decision trees can be highly accurate, however, they are prone to overfitting behavioural categories, that is, they are highly accurate at identifying training data but less so for unidentified data (Valletta et al., 2017). Automated RF models solve this problem by generating multiple decision trees from a subset of the available variables and a subset of the classified data, so are less subject to overfitting and have an increased accuracy (Cutler et al., 2007; Nathan et al., 2012; Valletta et al., 2017). However, inherent errors with RF modelling can occur such as incorrectly identifying or overlooking certain behaviours (Rast et al., 2020; Wang et al., 2015). Indeed, the accuracy of RF modelling has been reported to be as low as 0% for mountain lion (*Puma concolor*) behaviours such as grooming while their locomotory behaviours were identified with an accuracy above 90% (Wang et al., 2015). Graf et al. (2015) hypothesised that the erratic nature of grooming, which requires many postures and is conducted at varying frequencies, meant it was difficult to define using accelerometer metrics and hence, was often misidentified by RF models. Revising methods that can improve predictive accuracy is an important component of data processing that is often overlooked in ecological studies and has wide‐ranging implications that would benefit researchers by improving model outputs.

There are three main ways that have been described to change or improve the efficacy of RF modelling, and these are implemented during acceleration data processing before the RF models are fitted (Alvarenga et al., 2016; Pagano et al., 2017; Tatler et al., 2018). They are (i) increasing the number of calculated variables that improve the explanatory power and specificity in describing behaviours (Tatler et al., 2018; Wijers et al., 2018), (ii) increasing or decreasing the frequency of acceleration data recording (Fogarty et al., 2020; Wang et al., 2015) and (iii) ensuring that the training data incorporates a similar duration of each of the behaviours (here denoted ‘standardised duration’; Chen et al., 2004; Pagano et al., 2017; Wijers et al., 2018).

### 1.1 Choice of calculated variables

The variables calculated from accelerometer data that are used to generate an RF model can affect overall model accuracy (Tatler et al., 2018, Wijers et al., 2018). Many studies simply select commonly used variables, but do not investigate whether these generate the most accurate model (Fogarty et al., 2020; Venter et al., 2019). Variables typically consist of static and dynamic acceleration (Smith, 1997; Wilson et al., 2006), dynamic body acceleration (DBA) (Qasem et al., 2012; Wilson et al., 2020) and pitch and roll (Fehlmann et al., 2017; Nathan et al., 2012; Wilson et al., 2008). Potential extra variables might include the dominant power spectrum frequency and amplitude, and ratios of Vectoral Dynamic Body Acceleration (VeDBA) to dynamic acceleration (Fehlmann et al., 2017; Lush et al., 2018; Wang et al., 2015), to name just a few. While some metrics provide an instantaneous measurement of motion in one or up to three axes, the running standard error of any waveform indicates its amplitude and therefore the ‘size’ of the acceleration movement over time of a particular behaviour, which can therefore also be important in behaviour classifications (Laich et al., 2008; Nathan et al., 2012; Qasem et al., 2012; Smith, 1997).

### 1.2 Adjustment of accelerometer data frequency

Accelerometer data, while usually recorded at sub‐second sampling frequency (up to 140 Hz, Sur et al., 2017), are often summed or expressed as a mean over 1 or 2 s to provide summary metrics of movements (Lush et al., 2018; Pagano et al., 2017; Shepard, Wilson, Halsey, et al., 2008; Wijers et al., 2018). The use of these lower‐resolution recordings facilitates rapid processing of accelerometer data and can be an important consideration given computational power, battery life and the study duration and aims. However, higher sampling frequencies could provide more precise information for fast‐paced or high‐speed behaviours such as running (Chakravarty et al., 2019). Alternatively, aperiodic, or ‘slower’ behaviours such as feeding may, in fact, be represented better by an average over a few seconds (Alvarenga et al., 2016; Lush et al., 2018). Therefore, the inclusion of data recorded at different frequencies (via sub‐sampling or as a mean over time) has the potential to affect the accuracy and reliability of the RF model with which to predict behaviours (Alvarenga et al., 2016; Hounslow et al., 2019; Lush et al., 2018).

### 1.3 Standardised durations—balancing the duration of each behaviour in the training dataset

There is some evidence that RF models trained using datasets that have a larger number of examples of some behaviours than the others (i.e. they use every behaviour example collected and therefore have an ‘inconsistent’ duration of each in the dataset, e.g., an abundance of ‘resting’ behaviour), skew the predictions of behaviours in favour of the more abundant behaviour classification while less readily predicting infrequent behaviours (Chen et al., 2004; Smit et al., 2023). Behaviours that are hard to observe during calibrations, such as mating, may therefore be misclassified during wild animal behaviour predictions. This potential bias can be minimised by sub‐sampling abundant behaviours to generate a more ‘standardised’ duration distribution of behaviours in the training dataset (Pagano et al., 2017; Wijers et al., 2018).

This study aimed to examine how effective various RF models were at identifying behaviours when different aspects of the training data [(i) to (iii) above] were changed. These models were used to identify the behaviours of a model quadruped—free‐ranging domestic cats (*Felis catus*, hereafter ‘cats’). Cat behaviours were also manually identified using a decision tree to assess whether the RF models reliably identified the behaviours of free‐ranging animals. Cats were studied as they are a useful proxy for wild animal movement and behaviour research, in part because they are readily handled which facilitates device deployment, but also because they roam freely outdoors, replicating behaviours that might occur in wild cryptic terrestrial species. Furthermore, while accelerometers have been used to study cat activity previously (Andrews et al., 2015; Lascelles et al., 2008; Naik et al., 2018; Thomas et al., 2017), and some have identified cat behaviours from accelerometers (Kestler & Wilson, 2015; Watanabe et al., 2005; Watanabe & Takahashi, 2013), this research develops the use of RF models to efficiently and accurately process accelerometer data and identifies free‐ranging domestic cat behaviours in detail. We aim to provide a framework for other researchers using RF models for behaviour identification to improve model accuracy and generate reliable activity classifications.

## 2 MATERIALS AND METHODS

### 2.1 Animals and study sites

Nine adult domestic cats (4 females, 5 males; aged 6 months–8 years) which were housed inside (‘indoor cats’) at Mid Antrim Animal Sanctuary, Antrim, Northern Ireland, were collared and filmed to calibrate behaviours. Subsequently, five domestically owned cats (4 females, 1 male; aged 9 months–12 years, ‘outdoor cats’, see Table A1) that were free to roam outside their owners' houses were recruited in Northern Ireland and collared to identify their natural behaviours (see below and Appendix A for details).

### 2.2 Calibration of animal behaviours and accelerometer signals

Indoor cats were fitted with neck collars to which tri‐axial accelerometers (‘Daily Diary’: Wilson et al., 2008) recording at 40 Hz were affixed. Accelerometer data were synchronised with video footage of the cats and distinct behaviours were labelled (‘rest’, ‘walk’, ‘trot’, ‘run’, ‘collar shake’, ‘feed’ and ‘groom’) using bespoke software DDMT (Wildbyte technologies, http://wildbytetechnologies.com/software.html, Wilson et al., 2008, see Appendix A for details of synchronisation and accelerometer data sample extraction). Transitions between behaviours were not included in any behaviour sample. A total of 116 samples of calibrated behaviours that lasted at least 2 s (>80 accelerometer measurements) were extracted from the accelerometer data. This equated to 54.2 min of discrete observed behaviours (mean 361.14 ± 109.68 seconds per individual) with an average of 464.33 ± 345.01 seconds per behaviour (Table A2). Wang et al. (2015) and Nekaris et al. (2022) successfully identified animal behaviours using RF models trained using comparable sampling efforts so these observations should provide a robust training dataset.

#### 2.2.1 Development of a decision tree for behaviour identification

**FIGURE A1:**
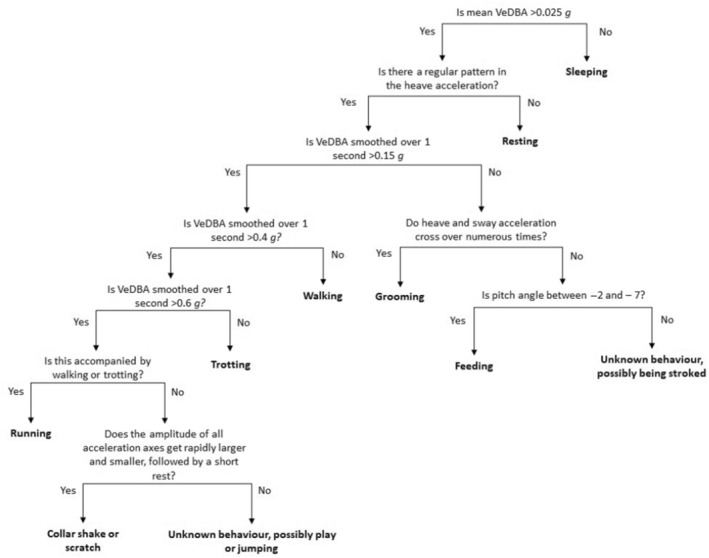
Decision tree for identifying free‐ranging domestic cat behaviours from tri‐axial acceleration, developed from manual calibrations of behaviours and accelerometer data using concurrent video recordings. ‘Sleep’ and ‘rest’ behaviours were characterised by long periods of inactivity. ‘Grooming’ behaviours included the cat licking its fur on all parts of its body including its back, tail and paws. ‘Feeding’ behaviour was solely from pellet food from a bowl. Locomotory behaviours, ‘walk’, ‘trot’ and ‘run’, were conducted in straight lines (with no corners) and characterised by the increasing speeds and different gaits. ‘Collar shake’ or scratch was typically conducted using a hind leg or a rotatory shake of the head. Unknown behaviours that were not defined but were observed included human interactions such as the cat being stroked and active behaviours such as jumping onto a high surface or playing with toys.

A decision tree for identifying behaviours from the accelerometer data was developed from the calibrated accelerometer signals. This was accomplished by an observer examining metrics derived from the examples of calibrated behaviour data. Distinguishing features were identified which were indicative of different movements, for example, a high VeDBA (sensu Qasem et al., 2012), changes in pitch, or patterns in the amplitude and frequency of the dynamic acceleration (see the decision tree Figure A1). The decision tree accuracy was tested by the observer using it to identify the calibrated samples of behaviours and calculate the percent that was correctly identified (Table A2).

### 2.3 Automated behaviour identification via RF modelling and model validation

#### 2.3.1 Generating the datasets for RF modelling

**FIGURE 1:**
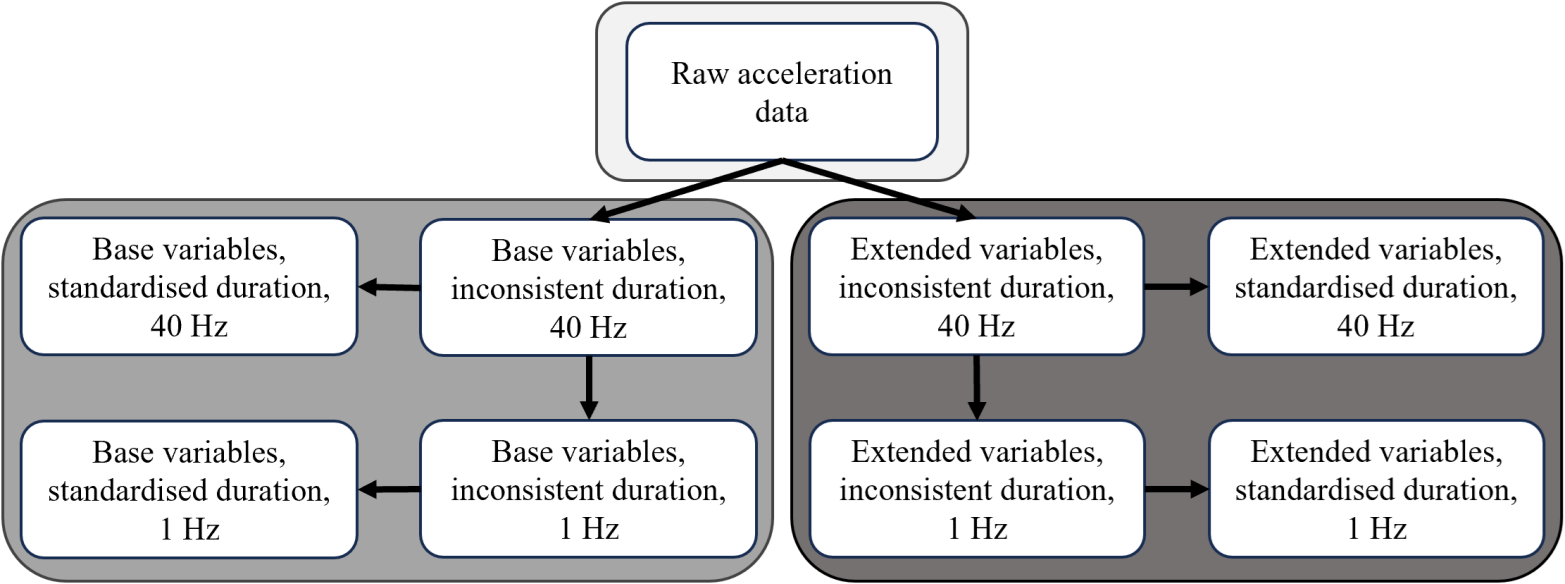
Development of datasets used for random forest modelling. Base datasets consisted of 13 ‘base’ variables including raw acceleration, static‐ and dynamic ‐acceleration, all in three axes, heave, surge and sway, plus VeDBA, smoothed VeDBA over 2 seconds, Pitch and Roll. ‘Extended’ datasets consisted of the base variables plus the standard error of raw and dynamic acceleration in all three axes, VeDBA and smoothed VeDBA. Data were collected at 40 Hz and the mean of each variable was also calculated over each second to generate datasets at 1 Hz. ‘Standardised duration’ datasets were derived from subsampling the ‘inconsistent duration’ 40 Hz and 1 Hz datasets, so each had a maximum of 60 seconds of any one behaviour, whereas ‘inconsistent duration’ datasets included all available behavioural samples.

From the labelled, video‐calibrated accelerometer data, a ‘base’ dataset of variables was calculated at 40 Hz. This included 13 variables; raw‐ (‘acc’), static‐ (‘st’) and dynamic acceleration (‘dy’), for all three axes: lateral (sway), vertical (heave) and sagittal (surge) (*x*, *y* and *z*, respectively). Vectoral dynamic body acceleration (VeDBA), smoothed VeDBA (‘VeDBAs’) over 2 s, ‘Pitch’ and ‘Roll’ were also calculated (definitions and equations for these variables are given in Appendix A and Table A3). A second ‘extended’ dataset at 40 Hz was generated by calculating eight further variables; the data from each behaviour were grouped and a running 2 s standard error of the variables was calculated (Table A4). Two further datasets were generated by calculating the mean values over 1 s for all the variables in that dataset, generating a base and an extended dataset at 1 Hz. Four more ‘standardised duration’ datasets were then derived from these by randomly subsampling the data to consist of a maximum of 60 s of each behaviour (rather than, e.g., over 2000 s of ‘rest’ behaviour) (Pagano et al., 2017). A time period of 60 s was chosen as most behaviours were recorded for at least this amount of time (Table A2), and this time period provided a large enough dataset to train and validate the models. Where less than 60 s of a certain behaviour occurred, 100% of these data were included in the analysis. These calculations generated eight training datasets (Figure 1) that were used to fit RF models for the identification of domestic cat behaviours.

#### 2.3.2 Generating the RF models

Using R software (version 3.4.0, R core team 2014) and the package *randomForest* (Breiman, 2001), RF models were generated from the eight datasets using a random sample of 60% of the calibrated data. To train each model, we fit 500 classification trees and used a random subset of three predictor variables for each split in the tree (Lush et al., 2018; Pagano et al., 2017). A minimum number of five data points was used during classification regressions and 10 during predictions (Breiman, 2001). These models were then used to predict the behaviours of the remaining 40% of the data. The most frequent prediction across all trees was selected as the final classification, which was then compared to the actual, video‐identified, behaviour (Breiman, 2001; Pagano et al., 2017). We calculated the ‘out‐of‐bag’ (OOB) error rate and the Gini Index for each model and evaluated the predictive accuracy of each model from the precision, recall and F‐measure of each behaviour (see Appendix A ‘Measuring the accuracy of RF models’). The Gini Index indicates the importance of a variable in improving the purity of behaviour classifications (Breiman, 2001; Christensen et al., 2023; Han et al., 2016). High F‐measures and low OOB error rates indicate good model accuracy, but a low OOB error rate combined with a low F‐measure indicates model overfitting, where the model can reliably classify data from the training dataset but not the validation dataset.

### 2.4 Free‐ranging cat behaviour identification

The five outdoor cats were fitted with collars bearing the same accelerometers (‘Daily Diary’, Wilson et al., 2008) set to record at 40 Hz (see Appendix A for details). Devices were fitted to hang under the chin of the cats and recorded for a total of 13.72 days (mean 2.74 ± 0.60 days per individual).

#### 2.4.1 Identification of free‐ranging cat behaviours via decision tree and RF models

The free‐ranging cat behaviours were first identified manually by a researcher examining the accelerometer data. Using the decision tree developed from the categorised data, they classified the behaviours of the first 15 min of each hour for all five cats, totalling 74.88 h of identified behaviours (mean 15.00 ± 3.61 h per cat). This was representative of the behaviours exhibited by the cats for the whole time they were collared (see Appendix A ‘Effects of identifying cat behaviours for 15 min per hour or the full time’). This method provided an accurate measure of the time cats spent engaged in the behaviours as a reference for comparison with the RF modelling.

Second, the behaviours of the free‐ranging cats were identified from their accelerometer data using the eight RF models developed from the training datasets, using the package *randomForest* (Breiman, 2001). To achieve this, their accelerometery data were used to calculate the same variables as those used to train the RF models, for example, the base variables were included when the RF models had been developed from base datasets (Table A4). The free‐ranging cat accelerometer variables were also calculated at either 40 Hz or using mean values over 1 second in the same way as the calibrated training data. The RF models were used to identify the behaviours at each instant in time (40 Hz or 1 Hz) using the 500 trees developed at each node and selected the most common outcome as the predicted behaviour. The total amount of time the cat spent on each behaviour was then summed. The time spent undertaking each behaviour was converted to a per cent of the time that the particular individual was collared.

### 2.5 Data analyses

Analyses were conducted using R (version 3.4.0, R core team 2014), with a statistical significance level of *p* < .05. Results are expressed as mean ± 1 standard error unless otherwise indicated. An intraclass correlation coefficient (ICC) was calculated with the *DescTools* package (Signorell, 2016) based on a single rating, absolute‐agreement, two‐way mixed effects model (Koo & Li, 2016) to compare the per cent of time cats spent on the behaviours predicted by the RF model with the per cent of time spent on the behaviours identified from the decision tree. The decision tree predictions of the behaviours were assumed to be the most precise method of behaviour identification as each behaviour signal could be compared to other examples of calibrated signals. The ICC model assessed the reliability of the two methods (the decision tree and one RF model in each case) for providing similar results in terms of behaviour frequency and rank. If the 95% confidence intervals of the ICC estimate were greater than 0.9, between 0.9 and 0.75, between 0.75 and 0.5 and less than 0.5, this was indicative of ‘excellent’, ‘good’, ‘moderate’ and ‘poor’ reliability, respectively (Koo & Li, 2016). In the first instance, all behaviours were included in this analysis before ‘rest’ behaviours were removed and the comparisons re‐run.

## 3 RESULTS

### 3.1 RF model accuracy for calibrated behaviours of indoor cats

The RF model that most accurately predicted known behaviours used the extended variables, with standardised duration of behaviours, at 40 Hz. In this model, the F‐measure was 0.96 ± 0.02 (Table 1) and the precision and recall were both above 0.95. The second most accurate model, with extended variables, inconsistent behaviour durations, at 40 Hz, had an F‐measure of 0.94 ± 0.05 and a precision and recall above 0.93. The accuracy of the RF models was lower when the mean of each second was calculated for the variables. The most accurate model, when using the mean over 1 second, was developed from the extended variables, with a standardised duration of behaviours. This had an F‐measure of 0.74 ± 0.05 and a precision and recall of 0.83 and 0.71 respectively. Thus, all datasets at 40 Hz generated more accurate models than those at 1 Hz, according to the F‐measure and the OOB error rate. In addition, the datasets with standardised durations of behaviours produced the models with the highest F‐measure for datasets at both 40 and 1 Hz.

**TABLE 1:** The F‐measure and out‐of‐bag (OOB) error rate (Breiman, 2001) of random forest models developed from datasets with a set of base or extended variables, a standardised or inconsistent duration of training behaviours, at 40 Hz or from the mean of each variable over 1 second.

	40 Hz		1 Hz	
	F‐measure	OOB	F‐measure	OOB
Base variables, inconsistent duration	0.89 ± 0.07	4.48%	0.66 ± 0.12	15.57%
Base variables, standardised duration	0.92 ± 0.03	9.04%	0.56 ± 0.11	31.79%
Extended variables, inconsistent duration	0.94 ± 0.05	2.18%	0.60 ± 0.15	13.67%
Extended variables, standardised duration	0.96 ± 0.02	5.26%	0.74 ± 0.05	28.49%

The OOB error rate was higher for models with standardised durations of behaviours than the models with inconsistent durations compared to those with the same variables and frequency. While a low OOB error rate combined with a low F‐measure can indicate model overfitting, the high F‐measures and higher OOB error rates seen here suggest the models with standardised durations of behaviours are less prone to overfitting than those with inconsistent durations of behaviours.

Prediction accuracy varied with behaviour. Using the most accurate model (with extended variables, standardised duration of behaviours, at 40 Hz), trot, run, shake, rest, feed and groom were all identified with an F‐measure above 0.92 but walk had an F‐measure of 0.88 (Table 2). The most accurate model at 1 Hz (with extended variables and standardised duration of behaviours) had more varying accuracy with different behaviours, most accurately predicting shake, feed, rest and run (F‐measures all over 0.8) but less accurately predicting groom (0.67), walk (0.58) and trot (0.58). In general, high‐frequency, fast‐paced behaviours (walk, trot, run and shake) were most accurately identified by models derived from the high‐frequency 40 Hz datasets. Across all 40 Hz models, high‐frequency behaviours were identified at an average F‐measure of 0.89 ± 0.14, whereas with models at 1 Hz, higher frequency behaviours were identified at an average F‐measure of 0.59 ± 0.24. Models derived from datasets at 1 Hz performed better at predicting low‐frequency behaviours (feed, groom, rest) than at predicting high‐frequency behaviours, with an average F‐measure of 0.71 ± 0.25.

**TABLE 2:** Precision, recall and F‐measure for random forest model testing of known cat behaviours, with the mean and standard error of the mean (SEM) for each model.

	Base variables, inconsistent durations, 40 Hz			Base variables, standardised durations, 40 Hz		
	Precision	Recall	F‐measure	Precision	Recall	F‐measure
Feed	0.99	0.91	0.95	0.97	0.99	0.98
Groom	1.00	0.88	0.93	0.97	0.96	0.96
Rest	0.99	0.99	0.99	0.95	0.88	0.92
Walk	0.87	0.91	0.89	0.82	0.79	0.80
Trot	0.54	0.47	0.50	0.83	0.88	0.85
Run	0.97	0.97	0.97	0.94	0.98	0.96
Shake	1.00	0.95	0.97	0.99	0.95	0.97
MEAN	0.91	0.87	0.89	0.92	0.92	0.92
SEM	0.06	0.07	0.07	0.03	0.03	0.03

	Base variables, inconsistent durations, 1 Hz			Base variables, standardised durations, 1 Hz		
	Precision	Recall	F‐measure	Precision	Recall	F‐measure
Feed	0.79	0.63	0.70	0.90	0.71	0.79
Groom	N/A	0.00	N/A	0.67	0.40	0.50
Rest	0.94	0.93	0.94	0.91	0.70	0.79
Walk	0.66	0.76	0.71	0.46	0.64	0.53
Trot	0.31	0.19	0.24	0.48	0.60	0.53
Run	0.68	0.78	0.72	0.77	0.80	0.78
Shake	N/A	0.00	N/A	0.00	0.00	0.00
MEAN	0.68	0.47	0.66	0.60	0.55	0.56
SEM	0.11	0.15	0.12	0.12	0.10	0.11

	Extended variables, inconsistent durations, 40 Hz			Extended variables, standardised durations, 40 Hz		
	Precision	Recall	F‐measure	Precision	Recall	F‐measure
Feed	1.00	1.00	1.00	1.00	1.00	1.00
Groom	1.00	0.96	0.98	0.96	0.99	0.98
Rest	1.00	1.00	1.00	0.98	0.90	0.93
Walk	0.94	0.94	0.94	0.88	0.88	0.88
Trot	0.64	0.62	0.63	0.89	0.96	0.93
Run	1.00	1.00	1.00	1.00	1.00	1.00
Shake	1.00	1.00	1.00	1.00	1.00	1.00
MEAN	0.94	0.93	0.94	0.96	0.96	0.96
SEM	0.05	0.05	0.05	0.02	0.02	0.02

	Extended variables, inconsistent durations, 1 Hz			Extended variables, standardised durations, 1 Hz		
	Precision	Recall	F‐measure	Precision	Recall	F‐measure
Feed	1.00	0.61	0.76	0.86	0.83	0.84
Groom	0.00	0.00	0.00	1.00	0.50	0.67
Rest	0.95	0.97	0.96	1.00	0.72	0.84
Walk	0.77	0.78	0.77	0.56	0.60	0.58
Trot	0.36	0.29	0.32	0.48	0.72	0.58
Run	0.81	0.78	0.79	0.89	0.89	0.89
Shake	N/A	0.00	N/A	1.00	0.67	0.80
MEAN	0.65	0.49	0.60	0.83	0.71	0.74
SEM	0.16	0.15	0.15	0.08	0.05	0.05

*Note*: N/A values occurred if no sample of the behaviour was correctly identified.

### 3.2 Identification of free‐ranging cat behaviours

#### 3.2.1 Reliability of RF behaviour identification

Cat behaviours identified by the observer using the decision tree showed cats spent 22.1 h (±15.2 min) a day resting on average, followed by walking (55.4 ± 19.9 min) and grooming (39.5 ± 4.6 min). This was followed by other locomotory behaviours (‘run’: 6.6 ± 3.9 min and ‘trot’: 5.0 ± 1.0 min), ‘collar shake’ (3.5 ± 0.6 min) and ‘feeding’ (2.7 ± 1.6 min). Validations of the decision tree showed the observer correctly identified cat behaviour 82.76% of the time (see Table A2).

**FIGURE 2:**
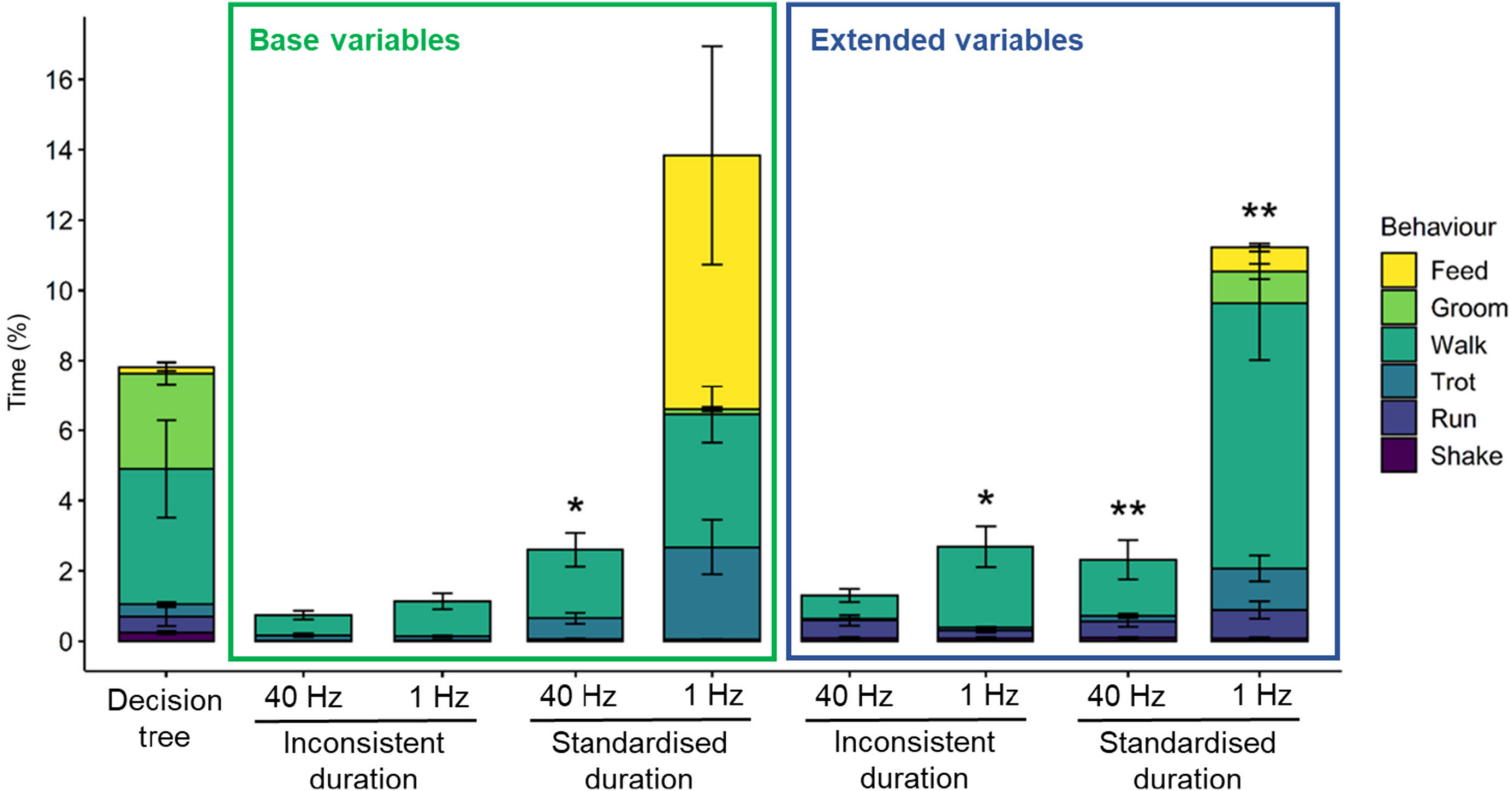
Mean and standard error of five free‐ranging domestic cats' per cent time spent on behaviours. Behaviours were identified from accelerometery data via a decision tree, and by random forest (RF) models derived from training datasets calibrated to behaviours via videoed accelerometery data of indoor cats. Definitions of each of the datasets used to develop the RF models can be found in Table A4. The time (per cent of the day) cats spent on behaviours predicted by each model are shown by colour (see Behaviour key). ‘Resting’ (not shown) made the total time to 100%. The model predictions were compared to the decision tree predictions through an interclass correlation coefficient (see the Statistics section for details) and good (**) and moderate (*) reliability is highlighted. The model that derived behaviours most similar to the behaviours identified using the decision tree was derived from extended variables, standardised durations of behaviours at 40 Hz.

Based on the ICC estimate for all behaviours, there was excellent reliability between the time spent on each behaviour that was identified by the decision tree and the RF models (range = 0.999–0.999). We note though, that the high proportion of identified ‘resting’ behaviour could have skewed the results towards this extremely high reliability as it comprised over 90% of the cat's behaviour. The reliability of the models decreased when ‘resting’ behaviour was removed from the analysis (detailed below) and likely more accurately established how reliable the models were at identifying behaviours other than ‘resting’. The two models with the highest degree of reliability were both derived from extended datasets with standardised duration of behaviours; this model at 40 Hz was the most reliable and had ‘good reliability’ (ICC of 0.756 ± 0.006), and this model at 1 Hz had ‘moderate to good reliability’ (ICC of 0.751 ± 0.006). These two models predicted different amounts of time the free‐ranging cats spent ‘walking’, ‘feeding’ and ‘grooming’ (Figure 2), where the 1 Hz model slightly overestimated the amount of time spent ‘walking’ compared to the decision tree estimate but predicted ‘feeding’ and ‘grooming’ more accurately than the 40 Hz model. Notably, the 40 Hz model predicted hardly any ‘feeding’ or ‘grooming’ behaviours (<0.04% of the time, Figure 2), and is likely therefore unfit for use to identify free‐roaming cat behaviours, despite its accuracy in predicting the behaviours in validations. Two of the remaining models, one with base variables, standardised duration of behaviours at 40 Hz and one with extended variables, inconsistent durations of behaviours at 1 Hz, had ‘moderate reliability’ (ICC between 0.641 and 0.748) compared to the decision tree‐identified behaviours. The remaining four RF models had ‘poor reliability’; these models had ICC values of less than 0.5 (see Table A5 for all ICC values and 95% CIs) (Koo & Li, 2016).

#### 3.2.2 Important variables for differentiating behaviours

**FIGURE 3:**
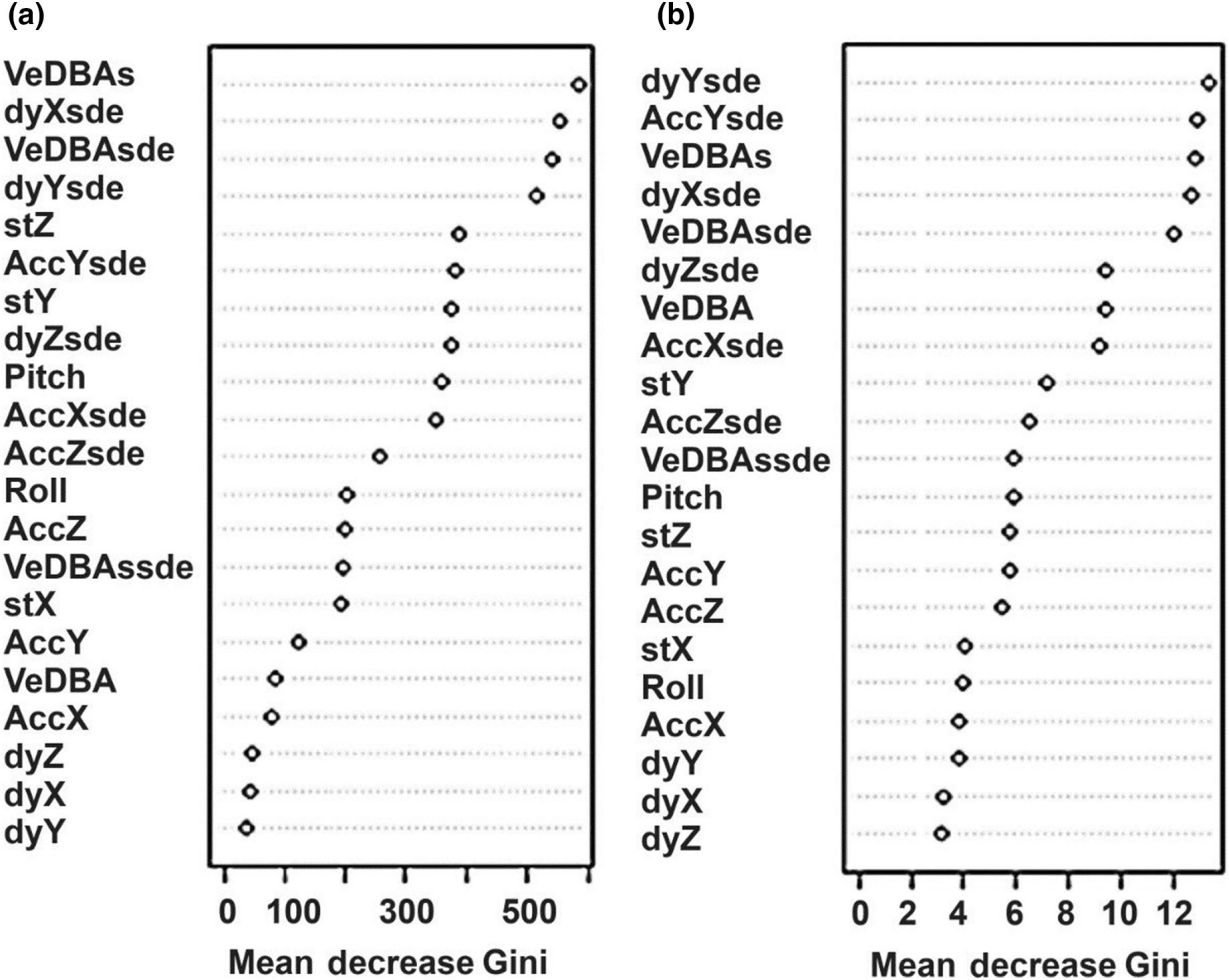
Relative importance of predictor variables for purity of domestic cat behaviour predictions based on the mean Gini index for (a) the 40 Hz and (b) 1 Hz model generated using extended variables with standardised durations of behaviours. Variable abbreviations are detailed in the methods and Table A2.

The variables that were most important for improving the purity of behaviour predictions were similar in the two models that were most accurate at identifying free‐ranging cat behaviours, both with extended variables, standardised durations of behaviours at 40 or 1 Hz. In fact, the top six variables were the same for both models, although in a different order (Figure 3), and at least six of the top 10 metrics were standard error variables and included the standard error of dynamic acceleration in all three axes. Both models also indicated that the dynamic acceleration of all three axes was the least important variable for improving node purity. The most important variables for the best model, at 40 Hz, were smoothed VeDBA, the standard error of the dynamic acceleration in the sway (*X*) axis, and then the standard error of VeDBA.

## 4 DISCUSSION

Identifying animal behaviours from accelerometery allows researchers to monitor cryptic species and study behaviours over a time span ranging from seconds to years (Nuijten et al., 2020; Wang et al., 2015; Wijers et al., 2018). Manual classification of long‐term studies of free‐ranging animals' behaviours can, however, be labour intensive (Hammond et al., 2016). Therefore, there has been increased interest in using supervised machine‐learning methods, such as RF modelling, that can increase the efficiency and accuracy of behaviour identifications. Model accuracy can vary substantially according to the species studied and the details of the methodology. RF models have been used to predict behaviours of a diverse range of species such as griffon vultures (*Gyps fulvus*) (Nathan et al., 2012), polar bears (*Ursus maritimus*) (Pagano et al., 2017) and sharks (lemon: *Negaproin brevirostris*; Brewster et al., 2018; white: *Carcharodon carcharias*; Gooden et al., 2024), but their accuracy of behaviour predictions can vary. Therefore, this study aimed to assess how accurately RF models predict behaviours when aspects of the data used to train the model were modified.

Our results indicate that data processing did make a difference in the model accuracy. Specifically, accuracy was highest when (i) the model included descriptive variables that were chosen as likely to differentiate between the behaviours (here demonstrated with extended datasets including standard error); (ii) the frequency of the data was highest or specifically matched the focal behaviour, such as to detect slower behaviours and (iii) the training data included a standardised duration for all behaviours. When our models were used to assess free‐roaming animal behaviours, the most reliable model during validation identified almost no ‘feeding’ and ‘grooming’ behaviours, rendering it unreliable and emphasising the advantage of validations of models for wild animal behaviours. Rast et al. (2020) similarly found a poor reliability of wild fox (*Vulpes vulpes*) behaviour predictions from RF models that were accurate during validations. Observations in the wild may not always be possible but monitoring individuals that were not included in the initial data collection would also be advantageous, either in captivity or those that are habituated. Techniques such as animal‐borne video cameras or direct observations should be used to validate model predictions in the wild or preferably to collect training data from wild animals that can be used to train the models (Gooden et al., 2024; Pagano et al., 2017). This, alongside adjustments to data pre‐processing, should increase the accuracy of RF model behaviour predictions and has wide‐ranging implications for many aspects of ecological research and conservation.

### 4.1 Effect of calculating standard error variables on model accuracy

The extended RF models derived using standard error variables had a higher accuracy than those with base variables (Table 1), demonstrating that variable selections should be critically considered to improve model accuracy. There are almost limitless variables that can be calculated, and indeed, studies have included between 8 and 128 variables in their models (Graf et al., 2015; Wijers et al., 2018), which have been further enhanced by other data, such as sound (Wijers et al., 2018), or multiple synchronised accelerometers in different locations (Tran et al., 2021). Smit et al. (2023) showed greater RF accuracy in identifying domestic cat behaviours when accelerometers were attached to a harness rather than a collar, however, harnesses can hinder movements or more easily become entangled if deployed in the wild. The selection and importance of different variables may depend on the species and its' behavioural characteristics or the behaviour of interest (Hathaway et al., 2023) as well as the computer power available—more variables require more processing power. Furthermore, we predict that if certain variables are demonstrably useful for a given species, these provide a good starting point for work on comparable species of different sizes or those that have similar locomotor modes, as seen in the similarity between useful predictor variables from RF models for pygmy goat (*Capra aegagrus hircus*) and Alpine ibex (*Capra ibex*) behaviours (Dickinson et al., 2021).

**FIGURE A2:**
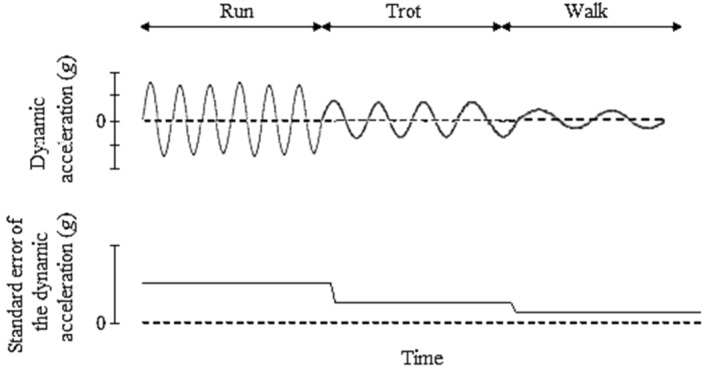
Diagrammatic representation of a single axis of dynamic acceleration and the standard error of the dynamic acceleration during three speeds of locomotion.

The high decrease in Gini found for standard error variables in the two most reliable models when classifying free‐ranging cat behaviours demonstrates that these are particularly useful for increasing the purity of behaviour differentiation (Figure A2). This concurs with Nathan et al. (2012) who note the usefulness of the standard deviation to identify griffon vulture (*Gyps fulvus*) behaviours. A running standard error calculated over an appropriate period provides a more constant measure of the overall size of the motion and represents the amplitude of the wave that will be consistently high for a high‐energy movement (Laich et al., 2008; Nathan et al., 2012) (Figure A2). Interestingly, and likely importantly, the dynamic acceleration in the heave, surge and sway axes were consistently ranked as the three least important variables. This could be due to the wave‐like form of dynamic acceleration that contains peaks and troughs that occur with each step giving a value that can be both positive and negative with appreciable variability over time (Laich et al., 2008). This inconsistency in the dynamic acceleration appears to hinder its use as a distinguishing factor between behaviours.

### 4.2 Effects of data frequency on model accuracy

Many studies identify behaviours from accelerometery data having taken a mean over 1 or 2 s (Fehlmann et al., 2017; Graf et al., 2015; Pagano et al., 2017) and Shepard, Wilson, Halsey, et al. (2008) suggest that variables should be ‘smoothed’ (i.e. taking a running mean) over a time period of one stroke cycle. Other studies have used smoothing periods of 3, 5 or 10 s (Campera et al., 2019; Chimienti et al., 2016; Lush et al., 2018) with varying effects on model predictive accuracy. Here, we investigated how smoothing period affected RF model accuracy by including and testing our 1 Hz datasets, however, a model derived at 40 Hz was most accurate for identifying cat behaviours during validation stages. The high‐frequency behaviours, such as ‘trotting’ and ‘running’, would have rapid oscillations in the accelerometer data and the 40 Hz dataset seems to have captured this detail. In contrast, the 1 Hz version of the same model had a low F‐measure but a good ICC reliability and provided a more accurate estimate of the time free‐ranging cats spent on the stationary behaviours, ‘feed’ and ‘groom’. We hypothesise that derivations of the mean over 1 Hz allowed a more accurate determination of stationary behaviours because these more accurately capture the motion of behaviours that are performed at a slower frequency. Slower or ‘aperiodic’ behaviours such as ‘grooming’ may be harder to identify from just a few points in the 40 Hz dataset due to the inconsistent nature of this behaviour (as noted by Graf et al., 2015 for Eurasian beavers, and Chakravarty et al., 2019). It may be indicative of the variety of grooming motion frequencies and postures adopted by cats to groom their whole body and, while these variations can be visually identified by the researcher using a decision tree, the RF models struggled to deal with the inconsistency in this behaviour. The period over which the mean is taken should also be considered, especially for larger animals that might have a slower stride frequency; for example, Alvarenga et al. (2016) found for sheep, that a mean calculated over 5 or 10 s led to a higher accuracy than over 3 s. Supporting this hypothesis, European pied flycatchers (*Ficedula hypoleuca*) catching prey at high speeds required a frequency of over 100 Hz for accurate identification whereas slower flight required 12.5 Hz (using the ‘rabc’ behaviour classification R package; Yu et al., 2023). Despite these behavioural considerations, study logistics including battery life will also influence decisions on the frequency of data collection. Certainly, our work indicates that the frequency of the data should be carefully evaluated when using RF modelling to identify specific animal behaviours accurately and indicates that taking a mean over 1 or 2 s would be particularly useful for identifying aperiodic behaviours, but the animal species and focal behaviour frequency should be considered and data processing conducted accordingly.

### 4.3 Effects of standardised durations of behaviours on model accuracy

An inconsistent duration of behaviours in the training dataset has been shown to bias model predictions towards the most abundant behaviours (Chen et al., 2004; Pagano et al., 2017) and, while every effort was made to record as many samples as possible of each cat behaviour, there was an abundance of ‘resting’ behaviour and relatively few examples of ‘groom’ and ‘shake’ behaviours in the data. These small sample sizes for specific behaviours did not appear to be a factor in behaviour identification accuracy, that is, they were not identified with any less precision or recall than other behaviours (Table A6). However, we did find that the models from datasets with standardised durations of each behaviour were more accurate than those with inconsistent durations of behaviours, which opposes the findings of Pagano et al. (2017) for polar bear behaviour identification who found uneven datasets were more accurate. While the higher OOB error rate and F‐measure seen for our models with standardised durations of each behaviour indicate a smaller chance of overfitting, this could also be due to the smaller datasample for these models; the OOB error rate is a percentage of incorrect classifications from the training data not used in each decision tree, so each ‘wrong’ classification had more effect. Nevertheless, there was good evidence that a standardised duration of behaviours increased model accuracy, so sub‐sampling over‐abundant behaviours to create a more even distribution does seem to be important in improving the predictive capabilities of RF modelling. Interestingly, the dataset size did not appear to influence overall accuracy scores; further testing of a 40 Hz dataset that was subsampled to a similar number of data points as the 1 Hz dataset (both with extended variables and standardised distributions of behaviours) showed that the 40 Hz dataset maintained a higher F‐measure (see Appendix A). This demonstrates that the absolute number of samples in the smaller 1 Hz dataset was not the driving factor in the lower F‐measures or OOB error rates.

## 5 CONCLUSIONS

RF models can be used to accurately predict animal behaviours using classified accelerometer data, but model accuracy can be improved via post‐data‐collect processing. Here, we show that high data frequencies, standardised durations of behaviours and extended variables improved model accuracy. The accuracy of models when identifying aperiodic behaviours, such as feeding and grooming, of animals in the wild may improve when using lower frequency data (means over 1 s) and suggests that the aperiodicity of focal behaviours should be taken into consideration when using RF modelling for identifying free‐ranging animal behaviours. The validation of behaviour predictions with known free‐ranging animal behaviours was important to reveal this trend and validations should also be prioritised in future studies to ensure wild animal behaviour predictions are accurate.

## AUTHOR CONTRIBUTIONS

**Carolyn E. Dunford:** Conceptualization (lead); data curation (lead); formal analysis (lead); investigation (lead); methodology (lead); validation (lead); visualization (lead); writing – original draft (lead); writing – review and editing (equal). **Nikki J. Marks:** Conceptualization (equal); funding acquisition (equal); project administration (equal); supervision (equal); writing – review and editing (equal). **Rory P. Wilson:** Conceptualization (equal); resources (equal); supervision (equal); writing – review and editing (equal). **D. Michael Scantlebury:** Conceptualization (equal); funding acquisition (equal); project administration (equal); supervision (equal); writing – original draft (equal); writing – review and editing (equal).

## CONFLICT OF INTEREST STATEMENT

The authors declare no conflict of interest.

## Data Availability

Data are available from the Dryad Digital Repository with the doi: 10.5061/dryad.q2bvq83sx.

## APPENDIX A

### Additional methods

#### Details of cats housed indoors for behaviour recording

Nine adult domestic cats (4 females, 5 males; aged 6 months–8 years) housed at Mid Antrim Animal Sanctuary, Antrim, Northern Ireland in rooms (2 m × 3 m) were studied in June and July 2017. Cats were free to move to an enclosed outside area (2 × 2 m). All individuals were either neutered or spayed and were certified as healthy by a veterinarian prior to participation in the study.

Cats were fitted with quick‐release collars (Breakaway buckle collar, Rogz Ltd. 2002/030628/07) to which a tri‐axial accelerometer (‘Daily Diary’, Wilson et al., 2008) recording at 40 Hz was attached. The total weight of the collar and logger was 25 g (less than 1% of the cats' body weight). Daily Diary loggers were fitted under the chin of the cat in line with the lateral (sway), sagittal (surge) and vertical (heave) body axes (Chakravarty et al., 2019). While wearing the collars, cats were filmed using a Sony Alpha a58 DSLR camera (Sony, Latin America, Inc.) for 15 min during the morning while a second researcher encouraged the cat to undertake different behaviours, such as running after a toy, or provided food to observe feeding behaviours. In addition, naturally occurring behaviours were observed, such as walking, trotting, resting, grooming and shaking the collar. These behaviours were selected as they accounted for much of the cat's (and other wild equivalent predator's) daily behaviours (Wilmers et al., 2017), are of ecological significance (Williams et al., 2014), and the repertoire can be indicative of welfare (Fuller et al., 2019). Each cat was filmed for 15 min to record the different behaviours it undertook.

#### Accelerometer data and video synchronisation

Accelerometer data and video footage from the indoor cats were synchronised using the timestamp of the data and video. To guard against any potential inaccuracies of their internal clocks, during the video, the collar was shaken up and down by the observers to create a distinct marking point in the accelerometer data that could be synchronised with the camera timestamp on the recording. Once the data were downloaded and loaded into DDMT software (Wildbyte technologies, http://wildbytetechnologies.com/software.html, Wilson et al., 2008), any offset that was required between the camera and the accelerometer was added to the accelerometer data. Distinct behaviours that lasted at least two seconds were selected on the video and identified in the accelerometer data via the corrected timestamp. Transitions between behaviours were not included in any behaviour sample. DDMT is a specialised accelerometer handling software, including facilitating the ‘labelling’ of behaviours that could then be extracted individually. This was conducted for all distinct identifiable behaviours within the video footage.

#### Random forest model generation

Random forest models use a subset of known behaviour data to ‘train’ the model to identify behaviours and use the remaining data subset to ‘test’ the model accuracy. Classification trees were built using a random ‘training’ subset of 60% of the data. The 500 trees grown from each training dataset were well above the recommended 300 trees required to acquire accurate results (Fehlmann et al., 2017).

To validate each model, it was used to predict the behaviours of the 40% of the dataset not used for training. For every measure of accelerometer data (at 40 Hz or 1 Hz), a behavioural prediction was made according to the classification from each of the 500 trees created from the training dataset. The most frequent prediction across all trees was selected as the final classification as the most likely behaviour, which was then compared to the actual, video‐identified, behaviour (Breiman, 2001; Pagano et al., 2017). We built a confusion matrix (a table of the frequencies of correct or incorrect behaviour predictions—Table A6) to assess the precision and recall of the model (Equations 1 and 2, Fehlmann et al., 2017) and evaluated the predictive abilities of the model based on the F‐measure (Equation 3) (see below for calculations of precision, recall and F‐measure). These metrics provide measures of model accuracy (Pagano et al., 2017).

The ‘out‐of‐bag’ (OOB) error rate (the per cent of events that were incorrectly classified from rows not included in each of the 500 decision trees; Breiman, 2001) and the Gini Index were calculated (Breiman, 2001; Fehlmann et al., 2017). The Gini Index indicates which variables improve the purity of behaviour classifications (Breiman, 2001; Christensen et al., 2023; Han et al., 2016) and was used to identify whether the ‘standard error variables’ were useful in behaviour identifications using the RF models.

#### Measuring the accuracy of random forest models

Precision, recall and F‐measure are based on the following categories of identification; true positive (TP) where the predicted behaviour is correctly identified as the actual behaviour (our example focal behaviour is resting, so, e.g., resting is identified as resting), true negative (TN) where the predicted behaviour is correctly identified as a different behaviour (not the focal behaviour, e.g. walking is identified as walking), false positive (FP) where the predicted behaviour is incorrectly identified as the focal behaviour (e.g. another behaviour is identified as resting) and false negative (FN) where the predicted behaviour is identified as an incorrect behaviour (e.g. resting is identified as grooming; Pagano et al., 2017).

(1)
$$\text{Precision}=\frac{\mathrm{TP}}{\mathrm{TP}+\mathrm{FP}}$$

(2)
$$\text{Recall}=\frac{\mathrm{TP}}{\mathrm{TP}+\mathrm{FN}}$$

(3)
$$F\;\text{measure}=2\times \frac{\text{precision}\times \text{recall}}{\text{precision}+\text{recall}}$$

If no FN results were obtained, such as when a certain behaviour was not selected in the random sample, then limited information could be gleaned as to how the focal behaviour was handled and thus an N/A result was returned for precision, and F‐measure. If TP and FP returned results of 0, such as when no behaviours were identified correctly, or no behaviours were identified as the focal behaviour incorrectly, then all samples of the focal behaviours were predicted as negative and returned N/A results for precision, and F‐measure. If the precision was 0 (when TP and FP were 0), the F‐measure could not be calculated and was returned as 0.

#### Generating the datasets for random forest modelling

Random forest models use variables to differentiate between behaviours. These are derived from the raw accelerometer data. The variables derived from the raw heave, surge and sway values (movements in orthogonal axes) of the identified accelerometer samples describe the animal's body motion and posture through acceleration and were selected as they describe the animals movement in different ways (Venter et al., 2019).

The variables calculated from the raw accelerometery data that constitute the base dataset were chosen based on previous accelerometery studies (Fehlmann et al., 2017; Shepard, Wilson, Halsey, et al., 2008; Shepard, Wilson, Quintana, et al., 2008; Watanabe & Takahashi, 2013; Wilson et al., 2006, 2008). This dataset can be used to train a random forest model which can then be used to identify behaviours from other accelerometer data.

A ‘base’ dataset of variables was calculated at 40 Hz. This comprised of 13 variables: raw acceleration (‘acc’), static acceleration (‘st’) and dynamic acceleration (‘dy’), all measured in three axes: sway, heave and surge (noted as *x*, *y* and *z*, respectively). The static acceleration represents the animal's posture, whereas the dynamic acceleration represents animal movements (Wilson et al., 2006). Vectoral dynamic body acceleration (‘VeDBA’), smoothed VeDBA (‘VeDBAs’), ‘Pitch’ and ‘Roll’ were also calculated (definitions and equations for these variables are provided in Tables A3 and A4).

A second ‘extended’ dataset at 40 Hz was generated by grouping the data from each behaviour and calculating a running 2‐second standard error of raw and dynamic acceleration in the sway, heave and surge axes, VeDBA, and smoothed VeDBA (Table A3). These further eight variables generated an ‘extended’ dataset that consisted of the base variables and the standard error variables (Table A4). These new variables examined the variation of each of the ‘active’ variables around the mean (Fehlmann et al., 2017; Laich et al., 2008; Watanabe & Takahashi, 2013).

Two further datasets were generated by calculating the mean values of the 40 Hz datasets over one second for all the variables in that dataset, generating a base and an extended dataset at 1 Hz. The frequency of the data has been shown to influence the reliability of behaviour identification when random forest modelling (Alvarenga et al., 2016).

Four more ‘standardised duration’ datasets were then derived, one from each of the base and extended datasets at 40 and 1 Hz. A long duration of examples of some behaviours can lead to a bias in the classification algorithm towards the more numerous behaviours (Chen et al., 2004). Therefore, a more even distribution of behaviours decreases the overestimation of the more numerous behaviours in training datasets. We used a similar method to Pagano et al. (2017), in which datasets of known behaviours were randomly subsampled to consist of a maximum of 60 s of each behaviour (rather than over 2000 s of rest behaviour, Table A2). Where less than 60 s of a certain behaviour occurred, 100% of these data was included in the analysis. In total, eight datasets were developed (Figure 1).

#### Free‐ranging cat data collection

Cat owners in Northern Ireland were contacted in 2016 and volunteered their animals to have their movements recorded. For the first 2 days of the study, the free‐roaming cats were fitted with ‘dummy collars’, which were the same size and weight as functioning collars but did not contain any devices. This allowed the cat to become accustomed to wearing the collar and the added weight of the devices. All the collars were fitted with a quick‐release clasp so that it would release if the cat became entangled. The collars were adjusted to fit each cat and allow two fingers to fit between the collar and the cat (Lord et al., 2010). Upon deployment, cats were monitored for 30 min to ensure there was no discomfort. Thereafter, owners monitored their cat's behaviour to watch for any signs of stress. In the trial, no signs of stress were observed in any of the individuals we measured, so all cats were included in the study. After two days, the dummy collars were then exchanged for one that carried a VHF radio transmitter (Tabcat homing tag © 2016 Loc8tor Ltd.) and an accelerometer tag (‘Daily Diary’, Wilson et al., 2008). The accelerometer was set to record at 40 Hz. The VHF was only used to find the collar if it became released from the cat (which happened on one occasion and the cat was fit with a replacement collar the same day). The total weight of the collar and loggers was 61 g, less than 1.5% of the body weight of any of the cats. Throughout the study, cats were allowed to move freely in and out of their owner's house via either a cat flap or being let in and out when required.

### Additional results

#### Free‐ranging cat behaviour identification from the decision tree

Through the validation process described in the text, the decision tree was accurate 82.76% of the time (Table A2). The behaviours identified from the accelerometer data using the decision tree found that the free‐ranging cats ‘rested’ for 22.12 h (92.17 ± 1.05%) each day, with the majority of the remaining time spent ‘walking’ (3.85 ± 1.38%) and ‘grooming’ (2.74 ± 0.32%). This was followed by other locomotory behaviours (‘run’: 0.46 ± 0.27% and ‘trot’: 0.35 ± 0.07%), then ‘collar shake’ (0.24 ± 0.04%) and ‘feeding’ (0.19 ± 0.11%).

#### Free‐ranging cat behaviours from RF models

‘Resting’ was the primary behaviour identified for all cats according to all models, where cats spent at least 21 hours ‘resting’ each day (Figure 2). Locomotion, particularly ‘walking’, was the second most commonly identified behaviour by the top models at 40 and 1 Hz, and comprised 5%–8% of each day. While there was almost no ‘feeding’ or ‘grooming’ identified by the top model at 40 Hz, the top model at 1 Hz predicted these behaviours more frequently but still underestimated both in comparison to the behaviours identified using the decision tree, particularly ‘grooming’ at 0.66 ± 0.07% compared to 2.74 ± 0.32% found using the decision tree. Grooming is a highly variable behaviour depending on which area of the body is groomed and may result in different accelerometer axes detecting motion, making it hard for the model to utilise differentiating variable features.

#### RF model accuracy—precision and recall

For the most accurate model, with extended variables and standardised durations at 40 Hz, the precision and recall for different behaviours ranged from 0.88 to 1.00 (Table 2). ‘Shake’, ‘feed’ and ‘run’ behaviours were the most reliably identified, whereas ‘walk’ behaviours had the lowest precision and recall and were commonly misclassified as ‘trot’ and vice versa. ‘Rest’ behaviours had high precision (0.98) but lower recall (0.90), whereas other behaviours were rarely classified as ‘resting’, but ‘rest’ behaviours were sometimes misclassified as ‘groom’, ‘feed’, ‘trot’, ‘run’ and most commonly as ‘walk’ (Table A6).

### Additional investigations

#### Effects of sample size on model accuracy

As supplementary analysis, we also investigated whether the large size of the datasets (where the 40 Hz datasets were larger than the 1 Hz dataset) might lead to higher accuracy due to the size of the dataset rather than the frequency of the data. We hypothesised that a larger dataset would lead to a higher accuracy of the models, not because the model necessarily identified behaviours more accurately at this higher frequency, but because the statistical power of these models from larger datasets would be greater (Thomas & Juanes, 1996) (e.g. the model with extended variables and standardised duration at 40 Hz contained 12,063 lines of data compared to the same dataset at 1 Hz that contained 320 lines of data). To investigate this, we examined whether this model at 40 Hz was more accurate than this model at 1 Hz due to the dataset size, by subsampling the 40 Hz dataset to include only 60 events (lines of data) per behaviour, rather than 60 s per behaviour. This led to a ‘subsampled dataset’ more comparable in size (*n* = 420) to the 1 Hz dataset. This demonstrated whether the dataset at 40 Hz was more accurate than at 1 Hz due to the detail of the behaviour waveform being maintained or the size of the dataset.

We used the subsampled dataset to identify cat behaviours and assessed the accuracy. The subsampled 60 event dataset developed a random forest model that had a precision of 0.82 ± 0.04, recall of 0.82 ± 0.07 and an F‐measure of 0.81 ± 0.05. This F‐measure was 6.7% more accurate than the random forest model trained using the extended dataset with standardised durations of behaviours at 1 Hz but was 14.9% less accurate than the same dataset at 40 Hz which suggests that the larger dataset size does affect the accuracy of the model, but also that the higher frequency dataset was still more accurate than the mean over one second.

In general, the higher frequency datasets (40 Hz) produced more accurate models than those derived from datasets at 1 Hz (derived from taking the mean of the variables over 1 s) (Table 1), however, when the dataset at 40 Hz was subsampled to include the same number of lines as the 1 Hz datasets, the modelling accuracy decreased. This shows that the size of the datasets can increase model accuracy when identifying behaviours. We found that the subsampled 60 event datasets were still more accurate at identifying behaviours than the model derived from a 1 Hz dataset which was similar in size (although this was not tested for free‐roaming cat data using the ICC reliability measure). This shows that the detail embedded in the accelerometery data recorded at 40 Hz is important when identifying behaviours and that taking a mean of the data can lose distinguishing features. This agrees with our above findings that behaviours that occur at a high frequency, such as locomotion in free‐ranging cats, are more reliably identified from a model derived from a higher frequency dataset, and that if quick locomotor behaviours are the focus of a study, high frequencies would likely provide the highest accuracy.

#### Effects of identifying cat behaviours for 15 minutes per hour or the full time

When recording animal behaviours, the amount of time that the animal is studied for can influence the outcome of behavioural predictions (Altmann, 1974) (i.e. 15 min/h or the full time). We therefore tested whether there was a difference in the amount of time spent on the different behaviours when they were identified for the first 15 min of each hour or for the whole time, the cat was collared. To test this, one of the five cats' behaviours was identified by the observer using the decision tree for the whole time it was collared (85.33 h).

The intraclass correlation coefficient (ICC) was calculated with the *DescTools* package (Signorell, 2016) based on a single rating, absolute‐agreement, two‐way mixed effects model (Koo & Li, 2016) and was used to assess the reliability of the observer identifying the behaviours of one cat for the first 15 min of each hour compared to identifying the whole time the cat was collared (as a per cent of the time identified). Analyses were conducted using R (version 3.4.0, R core team 2014). This showed whether the predictions of cat behaviours from the shorter observation times provided an accurate estimate of cat behaviours over the whole day.

There was ‘excellent reliability’ according to the ICC estimate (Koo & Li, 2016) between the time spent on each behaviour when identified by decision tree by an observer for 15 min per hour or for the whole time. The ICC estimate was 0.98 with a 95% confidence interval from 0.982 to 0.984, *F*(5, 5) = 59.6, *p* < .001, which shows that there was little difference in the time the cat was estimated to have spent on each behaviour whether the observer identified behaviours for 15 min or for the whole 60 min per hour and gives confidence to our predictions of cat behaviours.

**TABLE A1:** Details of free‐roaming domestic cats (*Felis catus*) fitted with accelerometer and GPS collars, with their age (years) and sex.

Cat ID	Age	Sex
A	10 months	F
B	Unknown	F
C	4	F
D	2	F
E	12	M

*Note*: ‘Unknown’ represents a rescued cat so the age was not confirmed.

**TABLE A2:** Duration (s) of video‐identified accelerometery data for each behaviour of nine indoor domestic cats.

Cat ID	Sex	Duration of behaviour (s)							Total
		Groom	Feed	Rest	Walk	Trot	Run	Shake	
1	F	7.20	0.00	66.88	38.88	2.93	21.08	3.80	140.75
2	F	3.58	2.88	592.18	339.40	12.05	0.00	0.00	950.08
3	F	0.00	0.00	299.80	36.53	0.00	0.00	0.00	336.33
4	F	0.00	0.00	489.13	0.00	0.00	0.00	0.00	489.13
5	M	0.00	0.00	6.38	60.83	61.58	15.80	3.35	147.93
6	M	2.30	54.10	40.70	10.45	0.00	11.63	0.00	119.18
7	M	0.00	10.00	129.90	35.85	0.00	0.00	0.00	175.75
8	M	0.00	0.00	35.48	11.73	8.03	2.30	0.00	57.53
9	M	0.00	0.00	831.35	0.00	2.28	0.00	0.00	833.63
Total		13.08	66.98	2491.78	533.65	86.85	50.80	7.15	3250.28
*N*		3	3	38	42	16	12	2	116
Decision tree accuracy (%)		66.67	66.67	86.84	95.24	62.50	58.33	100	82.76

*Note*: The number of behaviour samples longer than 2 s (*N*) and the accuracy of the decision tree for identifying the behaviours are also shown.

**TABLE A3:** Summary variables extracted from accelerometer data and used in random forest models to predict domestic cat behaviours.

Parameter	Notation	Calculation	Description	Reference
Raw acceleration (*g*)	${\mathrm{acc}}_{x}$, ${\mathrm{acc}}_{y}$, ${\mathrm{acc}}_{z}$		The acceleration measured in each orthogonal axis (*x*, *y* and *z*)	A
Static acceleration (*g*)	${\mathrm{st}}_{x}$, ${\mathrm{st}}_{y}$, ${\mathrm{st}}_{z}$	A running 2 s mean of ${\mathrm{acc}}_{x},{\mathrm{acc}}_{y},$ or ${\mathrm{acc}}_{z}$	The posture of the animal in each orthogonal axis (*x*, *y* and *z*)	B
Dynamic acceleration (*g*)	${\mathrm{dy}}_{x}$, ${\mathrm{dy}}_{y}$, ${\mathrm{dy}}_{z}$	acc−st for each axis, *x*, *y*, and *z*	The movement of the animal in each orthogonal axis (*x*, *y* and *z*)	B
Vectoral dynamic body acceleration (*g*)	VeDBA	$\sqrt{{{\mathrm{dy}}_{x}}^{2}+{{\mathrm{dy}}_{y}}^{2}+{{\mathrm{dy}}_{z}}^{2}}$	The overall body movement of the animal	B
Smoothed vectoral dynamic body acceleration (*g*)	VeDBAs	A running 2 s mean of VeDBA	The overall body movement of the animal without extreme peaks or troughs	C
Pitch	Pitch	$\text{asin}\left({\mathrm{st}}_{z}\right)$	Describes whether the animal is going up of down a slope	D
Roll	Roll	$\text{asin}\left({\mathrm{st}}_{x}\right)$	Describes whether an animal is banking or lying on its side	E
Standard error of raw acceleration	$\mathrm{SD}{\mathrm{acc}}_{x}$, S$D{\mathrm{acc}}_{y}$, $\mathrm{SD}{\mathrm{acc}}_{z}$	A running 2 s standard error of ${\mathrm{acc}}_{x},{\mathrm{acc}}_{y},$ or ${\mathrm{acc}}_{z}$	The magnitude of the raw acceleration in each orthogonal axis (*x*, *y* and *z*)	E
Standard error of dynamic acceleration	$\mathrm{SD}{\mathrm{dy}}_{x}$, $\mathrm{SD}{\mathrm{dy}}_{y}$, $\mathrm{SD}{\mathrm{dy}}_{z}$	A running 2 s standard error of ${\mathrm{dy}}_{x},{\mathrm{dy}}_{y},$ or ${\mathrm{dy}}_{z}$	The magnitude of the movement of the animal in each orthogonal axis (*x*, *y* and *z*)	D, F, H
Standard error of VeDBA	SDVeDBA	A running 2 s standard error of VeDBA	The magnitude of the overall body movement of the animal	D, F, H
Standard error of smoothed VeDBA	SDVeDBAs	A running 2 s standard error of VeDBAs	The magnitude of the overall body movement of the animal without extreme peaks or troughs	D, F, H

*Note*: Acceleration was measured in the sway (*x*), heave (*y*) and surge (*z*) axes. References are (A) Smith (1997) (B) Wilson et al. (2006), (C) Qasem et al. (2012), (D) Fehlmann et al. (2017), (E) Wilson et al. (2008), (F) Laich et al. (2008), (G) Watanabe and Takahashi (2013).

**TABLE A4:** Definitions of terminology for datasets.

Datasets	Definition
Base	A dataset consisting of the variables ${\mathrm{acc}}_{x},{\mathrm{acc}}_{y},{\mathrm{acc}}_{z},{\mathrm{st}}_{x},{\mathrm{st}}_{y},{\mathrm{st}}_{z},{\mathrm{dy}}_{x},{\mathrm{dy}}_{y},{\mathrm{dy}}_{z}$, VeDBA, VeDBAs,Pitch and Roll
Extended	A dataset consisting of the variables ${\mathrm{acc}}_{x},{\mathrm{acc}}_{y},{\mathrm{acc}}_{z},{\mathrm{st}}_{x},{\mathrm{st}}_{y},{\mathrm{st}}_{z},{\mathrm{dy}}_{x},{\mathrm{dy}}_{y},{\mathrm{dy}}_{z},\text{VeDBA}$,VeDBAs,Pitch, $\text{Roll},\mathrm{SD}{\mathrm{acc}}_{x},\mathrm{SD}{\mathrm{acc}}_{y},\mathrm{SD}{\mathrm{acc}}_{z}$, $\mathrm{SD}{\mathrm{dy}}_{x}$, $\mathrm{SD}{\mathrm{dy}}_{y},\mathrm{SD}{\mathrm{dy}}_{z}$, SDVeDBA and SDVeDBAs
Inconsistent duration	A dataset containing all available calibrated accelerometer behaviours over 2 s long
Standardised duration	A dataset with a maximum of 60 s of any one behaviour
40 Hz	A dataset of variables at 40 Hz
1 Hz	A dataset of the mean of the variables over 1 s

**TABLE A5:** The results of interclass correlation coefficient estimates and the 95% confidence intervals are based on a single rating, absolute‐agreement, two‐way mixed effects model.

Model	ICC estimate	*F*	*df*1	*df*2	*p* Value	Lower confidence interval	Upper confidence interval
Base variables, inconsistent duration, 40 Hz	0.247	1.328	29	29	.225	0.229	0.264
Base variables, standardised duration, 40 Hz	0.742	3.870	29	29	<.001	0.736	0.748
Base variables, inconsistent duration, 1 Hz	0.364	1.570	29	29	.114	0.349	0.379
Base variables, standardised duration, 1 Hz	0.079	1.090	29	29	.413	0.057	0.101
Extended variables, inconsistent duration, 40 Hz	0.148	1.173	29	29	.335	0.128	0.168
Extended variables, standardised duration, 40 Hz	0.756	4.100	29	29	<.001	0.750	0.762
Extended variables, inconsistent duration, 1 Hz	0.649	2.850	29	29	<.01	0.641	0.657
Extended variables, standardised duration, 1 Hz	0.751	4.010	29	29	<.001	0.745	0.757

*Note*: The agreement between the per cent time spent on behaviours each day by domestic cats was compared between behaviours that were predicted by each of the random forest models to behaviours predicted from accelerometery data identified by an observer using an ethogram.

**TABLE A6:** Confusion matrix for random forest model identification of cat feed, groom, rest, walk, trot, run and shake behaviours.

Observed behaviour	Predicted behaviour							
	Feed	Groom	Rest	Walk	Trot	Run	Shake	Total obs.
Feed	*966**	0	0	0	0	0	0	966
Groom	0	*166**	0	0	0	1	0	167
Rest	4	7	*867**	77	7	1	0	963
Walk	1	0	20	*887**	102	1	0	1011
Trot	0	0	0	34	*910**	0	0	944
Run	0	0	0	0	0	*718**	0	718
Shake	0	0	0	0	0	0	*57**	57
Total pred.	971	173	887	998	1019	721	57	4826

*Note*: The random forest model was trained using an extended set of variables (see Table A4), with a standardised duration of behaviours, using accelerometery data measured at 40 Hz. This matrix shows the accuracy of behaviour identification using the model. Predicted behaviours were identified using the model, and observed behaviours were identified from video recordings. Italicised and * values indicate correctly identified behaviours (true positive).

## References

[ece311380-bib-0001] Altmann, J. (1974). Observational study of behavior: Sampling methods. Behaviour, 49(3–4), 227–266. 10.1163/156853974X00534 4597405

[ece311380-bib-0002] Alvarenga, F. , Borges, I. , Palkovič, L. , Rodina, J. , Oddy, V. , & Dobos, R. (2016). Using a three‐axis accelerometer to identify and classify sheep behaviour at pasture. Applied Animal Behaviour Science, 181, 91–99. 10.1016/j.applanim.2016.05.026

[ece311380-bib-0003] Andrews, C. J. , Potter, M. A. , & Thomas, D. G. (2015). Quantification of activity in domestic cats (*Felis catus*) by accelerometery. Applied Animal Behaviour Science, 173, 17–21. 10.1016/j.applanim.2015.05.006

[ece311380-bib-0004] Barbour, K. , McClune, D. W. , Delahay, R. J. , Speakman, J. R. , McGowan, N. E. , Kostka, B. , Montgomery, I. W. , Marks, N. J. , & Scantlebury, D. M. (2019). No energetic cost of tuberculosis infection in European badgers (*Meles meles*). The Journal of Animal Ecology, 88(12), 1973–1985. 10.1111/1365-2656.13092 31411730

[ece311380-bib-0005] Bidder, O. R. , di Virgilio, A. , Hunter, J. S. , McInturff, A. , Gaynor, K. M. , Smith, A. M. , Dorcy, J. , & Rosell, F. (2020). Monitoring canid scent marking in space and time using a biologging and machine learning approach. Scientific Reports, 10, 588. 10.1038/s41598-019-57198-w 31953418 PMC6969016

[ece311380-bib-0006] Breiman, L. (2001). Random forests. Machine Learning, 45(1), 5–32. https://cran.r‐project.org/web/packages/randomForest/index.html

[ece311380-bib-0007] Brewster, L. , Dale, J. , Guttridge, T. , Gruber, S. , Hansell, A. , Elliott, M. , Cowx, I. , Whitney, N. , & Gleiss, A. (2018). Development and application of a machine learning algorithm for classification of elasmobranch behaviour from accelerometry data. Marine Biology, 165(4), 62. 10.1007/s00227-018-3318-y 29563648 PMC5842499

[ece311380-bib-0008] Bryce, C. M. , Dunford, C. E. , Pagano, A. M. , Wang, Y. , Borg, B. L. , Arthur, S. M. , & Williams, T. M. (2022). Environmental correlates of activity and energetics in a wide‐ranging social carnivore. Animal Biotelemetry, 10(1), 1–16.

[ece311380-bib-0009] Campera, M. , Balestri, M. , Chimienti, M. , Nijman, V. , Nekaris, K. A. I. , & Donati, G. (2019). Temporal niche separation between the two ecologically similar nocturnal primates *Avahi meridionalis* and *Lepilemur fleuretae* . Behavioral Ecology and Sociobiology, 73, 1–12. 10.1007/s00265-019-2664-1

[ece311380-bib-0010] Chakravarty, P. , Cozzi, G. , Ozgul, A. , & Aminian, K. (2019). A novel biomechanical approach for animal behaviour recognition using accelerometers. Methods in Ecology and Evolution, 10(6), 802–814. 10.1111/2041-210X.13172

[ece311380-bib-0011] Chen, C. , Liaw, A. , & Breiman, L. (2004). Using random forest to learn imbalanced data. University of California, Berkeley, 110(1–12), 24.

[ece311380-bib-0012] Chimienti, M. , Cornulier, T. , Owen, E. , Bolton, M. , Davies, I. M. , Travis, J. M. , & Scott, B. E. (2016). The use of an unsupervised learning approach for characterizing latent behaviors in accelerometer data. Ecology and Evolution, 6(3), 727–741. 10.1002/ece3.1914 26865961 PMC4739568

[ece311380-bib-0013] Christensen, C. , Bracken, A. M. , O'Riain, M. J. , Fehlmann, G. , Holton, M. , Hopkins, P. , King, A. J. , & Fürtbauer, I. (2023). Quantifying allo‐grooming in wild chacma baboons (*Papio ursinus*) using tri‐axial acceleration data and machine learning. Royal Society Open Science, 10(4), 221103. 37063984 10.1098/rsos.221103 PMC10090879

[ece311380-bib-0014] Cutler, D. R. , Edwards, T. C., Jr. , Beard, K. H. , Cutler, A. , Hess, K. T. , Gibson, J. , & Lawler, J. J. (2007). Random forests for classification in ecology. Ecology, 88(11), 2783–2792. 10.1890/07-0539.1 18051647

[ece311380-bib-0072] Dickinson, E. R. , Twining, J. P. , Wilson, R. , Stephens, P. A. , Westander, J. , Marks, N. , & Scantlebury, D. M. (2021). Limitations of using surrogates for behaviour classification of accelerometer data: refining methods using random forest models in Caprids. Movement Ecology, 9(1). 10.1186/s40462-021-00265-7 PMC8186069 34099067

[ece311380-bib-0015] Dunford, C. E. , Marks, N. J. , Wilmers, C. C. , Bryce, C. M. , Nickel, B. , Wolfe, L. L. , Scantlebury, D. M. , & Williams, T. M. (2020). Surviving in steep terrain: A lab‐to‐field assessment of locomotor costs for wild mountain lions (*Puma concolor*). Movement Ecology, 8, 1–12. 32782806 10.1186/s40462-020-00215-9 PMC7414561

[ece311380-bib-0016] Fehlmann, G. , O'Riain, M. J. , Hopkins, P. W. , O'Sullivan, J. , Holton, M. D. , Shepard, E. L. , & King, A. J. (2017). Identification of behaviours from accelerometer data in a wild social primate. Animal Biotelemetry, 5(1), 6. 10.1186/s40317-017-0121-3

[ece311380-bib-0017] Fogarty, E. S. , Swain, D. L. , Cronin, G. M. , Moraes, L. E. , & Trotter, M. (2020). Behaviour classification of extensively grazed sheep using machine learning. Computers and Electronics in Agriculture, 169, 105175. 10.1016/j.compag.2019.105175

[ece311380-bib-0018] Fuller, G. , Heintz, M. R. , & Allard, S. (2019). Validation and welfare assessment of flipper‐mounted time‐depth recorders for monitoring penguins in zoos and aquariums. Applied Animal Behaviour Science, 212, 114–122. 10.1016/j.applanim.2019.01.002

[ece311380-bib-0019] Gooden, A. , Clarke, T. M. , Meyer, L. , & Huveneers, C. (2024). Wildlife tourism has little energetic impact on the world's largest predatory shark. Animal Behaviour, 207, 247–265.

[ece311380-bib-0020] Graf, P. M. , Wilson, R. P. , Qasem, L. , Hackländer, K. , & Rosell, F. (2015). The use of acceleration to code for animal behaviours; a case study in free‐ranging Eurasian beavers Castor fiber. PLoS One, 10(8), e0136751. 10.1371/journal.pone.0136751 26317623 PMC4552556

[ece311380-bib-0021] Hammond, T. T. , Springthorpe, D. , Walsh, R. E. , & Berg‐Kirkpatrick, T. (2016). Using accelerometers to remotely and automatically characterize behavior in small animals. The Journal of Experimental Biology, 219(11), 1618–1624. 10.1242/jeb.136135 26994177

[ece311380-bib-0022] Han, H. , Guo, X. , & Yu, H. (2016). Variable selection using mean decrease accuracy and mean decrease gini based on random forest, *7th IEEE international conference on software engineering and service science* (*ICSESS*) (pp. 219–224).

[ece311380-bib-0023] Hathaway, A. , Campera, M. , Hedger, K. , Chimienti, M. , Adinda, E. , Ahmad, N. , Imron, M. A. , & Nekaris, K. A. I. (2023). Analysis of accelerometer data using random Forest models to classify the behavior of a wild nocturnal primate: Javan slow Loris (*Nycticebus javanicus*). Ecologies, 4(4), 636–653.

[ece311380-bib-0024] Hounslow, J. L. , Brewster, L. R. , Lear, K. O. , Guttridge, T. L. , Daly, R. , Whitney, N. M. , & Gleiss, A. C. (2019). Assessing the effects of sampling frequency on behavioural classification of accelerometer data. Journal of Experimental Marine Biology and Ecology, 512, 22–30. 10.1016/j.jembe.2018.12.003

[ece311380-bib-0025] Kestler, J. , & Wilson, M. (2015). Acceleration derived feral cat (*Felis catus*) behaviour during ground nesting bird‐breeding season on the island of Schiermonnikoog, *Doctoral dissertation*, *Van Hall Larenstein* .

[ece311380-bib-0026] Koo, T. K. , & Li, M. Y. (2016). A guideline of selecting and reporting intraclass correlation coefficients for reliability research. Journal of Chiropractic Medicine, 15(2), 155–163. 10.1016/j.jcm.2016.02.012 27330520 PMC4913118

[ece311380-bib-0027] Laich, A. G. , Wilson, R. P. , Quintana, F. , & Shepard, E. L. (2008). Identification of imperial cormorant Phalacrocorax atriceps behaviour using accelerometers. Endangered Species Research, 10, 29–37. 10.3354/esr00091

[ece311380-bib-0028] Lascelles, B. D. X. , Hansen, B. D. , Thomson, A. , Pierce, C. C. , Boland, E. , & Smith, E. S. (2008). Evaluation of a digitally integrated accelerometer‐based activity monitor for the measurement of activity in cats. Veterinary Anaesthesia and Analgesia, 35(2), 173–183. 10.1111/j.1467-2995.2007.00367.x 17927675

[ece311380-bib-0029] Li, X. (2013). Using “random forest” for classification and regression. Chinese Journal of Applied Entomology, 50(4), 1190–1197.

[ece311380-bib-0030] Lord, L. K. , Griffin, B. , Slater, M. R. , & Levy, J. K. (2010). Evaluation of collars and microchips for visual and permanent identification of pet cats. Journal of the American Veterinary Medical Association, 237(4), 387–394. 10.2460/javma.237.4.387 20707748

[ece311380-bib-0031] Lush, L. , Ellwood, S. , Markham, A. , Ward, A. , & Wheeler, P. (2016). Use of tri‐axial accelerometers to assess terrestrial mammal behaviour in the wild. Journal of Zoology, 298(4), 257–265.

[ece311380-bib-0032] Lush, L. , Wilson, R. P. , Holton, M. D. , Hopkins, P. , Marsden, K. A. , Chadwick, D. R. , & King, A. J. (2018). Classification of sheep urination events using accelerometers to aid improved measurements of livestock contributions to nitrous oxide emissions. Computers and Electronics in Agriculture, 150(C), 170–177.

[ece311380-bib-0033] McClune, D. W. , Marks, N. J. , Wilson, R. P. , Houghton, J. D. , Montgomery, I. W. , McGowan, N. E. , Gormley, E. , & Scantlebury, M. (2014). Tri‐axial accelerometers quantify behaviour in the Eurasian badger (*Meles meles*): Towards an automated interpretation of field data. Animal Biotelemetry, 2(1), 5. 10.1186/2050-3385-2-5

[ece311380-bib-0034] McGowan, N. E. , Marks, N. J. , Maule, A. G. , Schmidt‐Küntzel, A. , Marker, L. L. , & Scantlebury, D. M. (2022). Categorising cheetah behaviour using tri‐axial accelerometer data loggers: A comparison of model resolution and data logger performance. Movement Ecology, 10(1), 1–17. 35123592 10.1186/s40462-022-00305-w PMC8818224

[ece311380-bib-0035] Migli, D. , Astaras, C. , Boutsis, G. , Diakou, A. , Karantanis, N. E. , & Youlatos, D. (2021). Spatial ecology and diel activity of European wildcat (*Felis silvestris*) in a protected lowland area in northern Greece. Animals, 11(11), 3030. 34827762 10.3390/ani11113030 PMC8614438

[ece311380-bib-0036] Naik, R. , Witzel, A. , Albright, J. D. , Siegfried, K. , Gruen, M. E. , Thomson, A. , Price, J. , & Lascelles, B. D. X. (2018). Pilot study evaluating the effect of feeding method on overall activity of neutered indoor pet cats. Journal of Veterinary Behavior, 25, 9–13. 10.1016/j.jveb.2018.02.001

[ece311380-bib-0037] Nathan, R. , Spiegel, O. , Fortmann‐Roe, S. , Harel, R. , Wikelski, M. , & Getz, W. M. (2012). Using tri‐axial acceleration data to identify behavioural modes of free‐ranging animals: General concepts and tools illustrated for griffon vultures. The Journal of Experimental Biology, 215(6), 986–996. 10.1242/jeb.058602 22357592 PMC3284320

[ece311380-bib-0038] Nekaris, K. A.‐I. , Campera, M. , Chimienti, M. , Murray, C. , Balestri, M. , & Showell, Z. (2022). Training in the dark: Using target training for non‐invasive application and validation of accelerometer devices for an endangered primate (*Nycticebus bengalensis*). Animals, 12(4), 411. 35203119 10.3390/ani12040411 PMC8868541

[ece311380-bib-0039] Nuijten, R. , Prins, E. F. , Lammers, J. , Mager, C. , & Nolet, B. A. (2020). Calibrating tri‐axial accelerometers for remote behavioural observations in Bewick's swans. Journal of Zoo and Aquarium Research, 8(4), 231–238. 10.19227/jzar.v8i4.522

[ece311380-bib-0040] Pagano, A. M. , Rode, K. D. , Cutting, A. , Owen, M. , Jensen, S. , Ware, J. , Robbins, C. , Durner, G. M. , Atwood, T. C. , & Obbard, M. (2017). Using tri‐axial accelerometers to identify wild polar bear behaviors. Endangered Species Research, 32, 19–33. 10.3354/esr00779

[ece311380-bib-0041] Pagano, A. M. , & Williams, T. M. (2019). Estimating the energy expenditure of free‐ranging polar bears using tri‐axial accelerometers: A validation with doubly labelled water. Ecology and Evolution, 9(7), 4210–4219. 10.1002/ece3.5053 31015999 PMC6468055

[ece311380-bib-0042] Qasem, L. , Cardew, A. , Wilson, A. , Griffiths, I. , Halsey, L. G. , Shepard, E. L. , Gleiss, A. C. , & Wilson, R. (2012). Tri‐axial dynamic acceleration as a proxy for animal energy expenditure; should we be summing values or calculating the vector? PLoS One, 7(2), e31187. 10.1371/journal.pone.0031187 22363576 PMC3281952

[ece311380-bib-0043] Rast, W. , Kimmig, S. E. , Giese, L. , & Berger, A. (2020). Machine learning goes wild: Using data from captive individuals to infer wildlife behaviours. PLoS One, 15(5), e0227317. 10.1371/journal.pone.0227317 32369485 PMC7200095

[ece311380-bib-0044] Riaboff, L. , Aubin, S. , Bédère, N. , Couvreur, S. , Madouasse, A. , Goumand, E. , Chauvin, A. , & Plantier, G. (2019). Evaluation of pre‐processing methods for the prediction of cattle behaviour from accelerometer data. Computers and Electronics in Agriculture, 165, 104961. 10.1016/j.compag.2019.104961

[ece311380-bib-0045] Shepard, E. L. , Wilson, R. P. , Halsey, L. G. , Quintana, F. , Laich, A. G. , Gleiss, A. C. , Liebsch, N. , Myers, A. E. , & Norman, B. (2008). Derivation of body motion via appropriate smoothing of acceleration data. Aquatic Biology, 4(3), 235–241. 10.3354/ab00104

[ece311380-bib-0046] Shepard, E. L. , Wilson, R. P. , Quintana, F. , Laich, A. G. , Liebsch, N. , Albareda, D. A. , Halsey, L. G. , Gleiss, A. , Morgan, D. T. , & Myers, A. E. (2008). Identification of animal movement patterns using tri‐axial accelerometry. Endangered Species Research, 10, 47–60. 10.3354/esr00084

[ece311380-bib-0047] Shuert, C. R. , Pomeroy, P. P. , & Twiss, S. D. (2018). Assessing the utility and limitations of accelerometers and machine learning approaches in classifying behaviour during lactation in a phocid seal. Animal Biotelemetry, 6(1), 14. 10.1186/s40317-018-0158-y

[ece311380-bib-0048] Signorell, A. (2016). DescTools: Tools for descriptive statistics, *R package version 0*.*99*, 18. http://CRAN.R‐project.org/package=DescTools

[ece311380-bib-0049] Smit, M. , Ikurior, S. J. , Corner‐Thomas, R. A. , Andrews, C. J. , Draganova, I. , & Thomas, D. G. (2023). The use of triaxial accelerometers and machine learning algorithms for behavioural identification in domestic cats (*Felis catus*): A validation study. Sensors, 23(16), 7165. 10.3390/s23167165 37631701 PMC10458840

[ece311380-bib-0050] Smith, S. W. (1997). The scientist and engineer's guide to digital signal processing. California Technical Publishing, 35.

[ece311380-bib-0051] Soltis, J. , Wilson, R. P. , Douglas‐Hamilton, I. , Vollrath, F. , King, L. E. , & Savage, A. (2012). Accelerometers in collars identify behavioral states in captive African elephants *Loxodonta africana* . Endangered Species Research, 18(3), 255–263. 10.3354/esr00452

[ece311380-bib-0052] Sur, M. , Suffredini, T. , Wessells, S. M. , Bloom, P. H. , Lanzone, M. , Blackshire, S. , Sridhar, S. , & Katzner, T. (2017). Improved supervised classification of accelerometry data to distinguish behaviors of soaring birds. PLoS One, 12(4), e0174785. 10.1371/journal.pone.0174785 28403159 PMC5389810

[ece311380-bib-0053] Tatler, J. , Cassey, P. , & Prowse, T. A. A. (2018). High accuracy at low frequency: Detailed behavioural classification from accelerometer data. Journal of Experimental Biology, 221(23), jeb184085. 10.1242/jeb.184085 30322979

[ece311380-bib-0054] Thomas, D. G. , Post, M. , & Bosch, G. (2017). The effect of changing the moisture levels of dry extruded and wet canned diets on physical activity in cats. Journal of Nutritional Science, 6, e9. 10.1017/jns.2017.9 28620484 PMC5465855

[ece311380-bib-0055] Thomas, L. , & Juanes, F. (1996). The importance of statistical power analysis: An example from animal behaviour. Animal Behaviour, 52(4), 856–859. 10.1006/anbe.1996.0232

[ece311380-bib-0056] Tran, D. N. , Nguyen, T. N. , Khanh, P. C. P. , & Tran, D. T. (2021). An iot‐based design using accelerometers in animal behavior recognition systems. IEEE Sensors Journal, 22(18), 17515–17528.

[ece311380-bib-0057] Ullmann, W. , Fischer, C. , Kramer‐Schadt, S. , Pirhofer Walzl, K. , Eccard, J. A. , Wevers, J. P. , Hardert, A. , Sliwinski, K. , Crawford, M. S. , Glemnitz, M. , & Blaum, N. (2023). The secret life of wild animals revealed by accelerometer data: How landscape diversity and seasonality influence the behavioural types of European hares. Landscape Ecology, 38, 1–15.37362204

[ece311380-bib-0058] Valletta, J. J. , Torney, C. , Kings, M. , Thornton, A. , & Madden, J. (2017). Applications of machine learning in animal behaviour studies. Animal Behaviour, 124, 203–220. 10.1016/j.anbehav.2016.12.005

[ece311380-bib-0059] Venter, Z. S. , Hawkins, H. , & Cramer, M. D. (2019). Cattle don't care: Animal behaviour is similar regardless of grazing management in grasslands. Agriculture, Ecosystems & Environment, 272, 175–187. 10.1016/j.agee.2018.11.023

[ece311380-bib-0060] Wang, G. (2019). Machine learning for inferring animal behavior from location and movement data. Ecological Informatics, 49, 69–76. 10.1016/j.ecoinf.2018.12.002

[ece311380-bib-0061] Wang, Y. , Nickel, B. , Rutishauser, M. , Bryce, C. M. , Williams, T. M. , Elkaim, G. , & Wilmers, C. C. (2015). Movement, resting, and attack behaviors of wild pumas are revealed by tri‐axial accelerometer measurements. Movement Ecology, 3(1), 2. 10.1186/s40462-015-0030-0 25709837 PMC4337468

[ece311380-bib-0062] Watanabe, S. , Izawa, M. , Kato, A. , Ropert‐Coudert, Y. , & Naito, Y. (2005). A new technique for monitoring the detailed behaviour of terrestrial animals: A case study with the domestic cat. Applied Animal Behaviour Science, 94(1), 117–131. 10.1016/j.applanim.2005.01.010

[ece311380-bib-0063] Watanabe, Y. Y. , & Takahashi, A. (2013). Linking animal‐borne video to accelerometers reveals prey capture variability. Proceedings of the National Academy of Sciences of the United States of America, 110(6), 2199–2204. 10.1073/pnas.1216244110 23341596 PMC3568313

[ece311380-bib-0064] Wijers, M. , Trethowan, P. , Markham, A. , Du Preez, B. , Chamaillé‐Jammes, S. , Loveridge, A. , & Macdonald, D. (2018). Listening to lions: Animal‐borne acoustic sensors improve bio‐logger calibration and behaviour classification performance. Frontiers in Ecology and Evolution, 6, 171. 10.3389/fevo.2018.00171

[ece311380-bib-0065] Williams, T. M. , Wolfe, L. , Davis, T. , Kendall, T. , Richter, B. , Wang, Y. , Bryce, C. , Elkaim, G. H. , & Wilmers, C. C. (2014). Mammalian energetics: Instantaneous energetics of puma kills reveal advantage of felid sneak attacks. Science (New York, N.Y.), 346(6205), 81–85. 10.1126/science.1254885 25278610

[ece311380-bib-0066] Wilmers, C. C. , Isbell, L. A. , Suraci, J. P. , & Williams, T. M. (2017). Energetics‐informed behavioral states reveal the drive to kill in African leopards. Ecosphere, 8(6), e01850. 10.1002/ecs2.1850

[ece311380-bib-0067] Wilmers, C. C. , Nickel, B. , Bryce, C. M. , Smith, J. A. , Wheat, R. E. , & Yovovich, V. (2015). The golden age of bio‐logging: How animal‐borne sensors are advancing the frontiers of ecology. Ecology, 96(7), 1741–1753. 10.1890/14-1401.1 26378296

[ece311380-bib-0068] Wilson, R. P. , Börger, L. , Holton, M. D. , Scantlebury, D. M. , Gómez‐Laich, A. , Quintana, F. , Rosell, F. , Graf, P. M. , Williams, H. , Gunner, R. , Hopkins, L. , Marks, N. , Geraldi, N. R. , Duarte, C. M. , Scott, R. , Strano, M. S. , Robotka, H. , Eizaguirre, C. , Fahlman, A. , & Shepard, E. L. (2020). Estimates for energy expenditure in free‐living animals using acceleration proxies: A reappraisal. Journal of Animal Ecology, 89(1), 161–172. 31173339 10.1111/1365-2656.13040 PMC7030956

[ece311380-bib-0069] Wilson, R. P. , Shepard, E. , & Liebsch, N. (2008). Prying into the intimate details of animal lives: Use of a daily diary on animals. Endangered Species Research, 4(1–2), 123–137. 10.3354/esr00064

[ece311380-bib-0070] Wilson, R. P. , White, C. R. , Quintana, F. , Halsey, L. G. , Liebsch, N. , Martin, G. R. , & Butler, P. J. (2006). Moving towards acceleration for estimates of activity‐specific metabolic rate in free‐living animals: The case of the cormorant. Journal of Animal Ecology, 75(5), 1081–1090. 10.1111/j.1365-2656.2006.01127.x 16922843

[ece311380-bib-0071] Yu, H. , Muijres, F. T. , te Lindert, J. S. , Hedenström, A. , & Henningsson, P. (2023). Accelerometer sampling requirements for animal behaviour classification and estimation of energy expenditure. Animal Biotelemetry, 11(1), 28.

